# Transitioning of the Chemical Industry Toward a Net‐Zero Carbon Dioxide Emission Path

**DOI:** 10.1002/anie.202522234

**Published:** 2025-12-12

**Authors:** Ferdi Schüth, Stephan A. Schunk

**Affiliations:** ^1^ Max‐Planck‐Institut für Kohlenforschung Kaiser‐Wilhelm‐Platz 1 45470 Mülheim an der Ruhr Germany; ^2^ hte GmbH Kurpfalzring 103 60123 Heidelberg Germany; ^3^ BASF SE Carl‐Bosch‐Straße 38 67056 Ludwigshafen Germany; ^4^ Institute for Chemical Technology University Leipzig Linnéstraße 3 04103 Leipzig Germany

**Keywords:** Chemical industry, Energy demand, Greenhouse gas, Hydrogen, Methanol

## Abstract

Emissions from the chemical industry, both for energy and use of raw materials, account for approximately 6% of man‐made greenhouse gas emissions. In order to keep global warming at acceptable levels, these emissions—as all other emissions—have to be drastically reduced. One way to do this is the elimination of fossil feedstock from chemical production and meeting the energy demand from renewable resources. This contribution shows that the essential elements are already available at scale to provide C_1_‐building blocks, olefins, aromatics, and ammonia as the key base chemicals. Methanol can be produced from CO_2_ and renewable hydrogen, olefins from the methanol‐to‐olefins and related processes, for aromatics, the methanol‐to‐aromatics process is available, supplemented by biomass and recycled polymers as feedstock, and also for ammonia process concepts with a strongly reduced greenhouse gas footprint are available. Current hurdles are the partly unattractive economic boundary conditions and the rate at which a change in the feedstock situation can be achieved. Moreover, high amounts of renewable energy are required, which accounts for about half of the current global electricity production.

## Graphical abstract

One way to reduce the greenhouse gas footprint of the chemical industry to almost zero would be the production of the platform molecules C1‐compounds, olefins, aromatics, and ammonia by non‐fossil pathways. The rest of the chemical production chains could be essentially left unchanged, provided the process energy input is renewable. The biggest challenges include sourcing the massive amounts of hydrogen needed and bringing the cost further down.

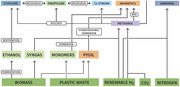

## 1 Introduction: The Value Chains of the Chemical Industry

Growing concerns caused by global warming exert pressure on many industrial sectors to reduce their greenhouse gas emissions (in the following also labelled as CO_2_ emissions, meaning the CO_2_ equivalent of all greenhouse gases). While energy conversion processes relying on fossil resources, such as coal, oil, or gas, are clearly the biggest source of CO_2_ emissions, other sectors also contribute substantially, and emissions from these sectors are often more difficult to abate. The chemical industry, in the combined demand for energy purposes and direct process emissions, accounts for close to 6% (data for 2016)^[^ ^1^ ^]^ of the global greenhouse gas emissions, making it an important sector to address.

The chemical industry has a diverse product portfolio, estimated at several tens of thousands of different chemical entities, with exact numbers varying from source to source.^[^ ^2^, ^3^ ^]^ To become greenhouse‐gas neutral, one could completely redesign the synthesis of any of these, but doing this would mean abandoning 150 years of development work of the chemical industry, replacing optimized and atom‐efficient synthesis routes, and vast amounts of capital would be required for investment into new assets. Alternatively, if one could use the current feedstock base, but produced in a greenhouse‐gas‐neutral manner, one could essentially leave downstream processes as they are today, if process energy input in these processes were sourced from renewable energies. In such a scenario, in some domains, there would remain residual greenhouse gas emissions, but a very high fraction of the CO_2_ footprint of chemical production could be removed.

In a real‐world scenario, a hybrid of both approaches—complete redesign of all value chains or basing current production on a zero‐greenhouse gas emissions basis—would most probably be realized, since both approaches have their value and could contribute to an industry transition. Moreover, new products with similar functionality, but low or zero carbon dioxide footprint might replace existing ones. The focus of this contribution, however, is on a CO_2_‐neutral feedstock base of chemical production as one base scenario. We propose that the essential technology components are available, albeit in some cases at higher cost or not yet at scale. Several of the aspects of this contribution are also covered in an excellent technology study published by DECHEMA,^[^ ^4^ ^]^ and a subsequent report of the Chemistry4Climate study, focused on the German situation.^[^ ^5^ ^]^ A different perspective—not necessarily with different overall conclusions—has recently been given by Vogt and Weckhuysen, who looked at a potential refinery of the future.^[^ ^6^ ^]^ The production of CO_2_‐neutral fuels is the focus of that contribution, but functional molecules and polymers are also addressed. In a future energy/chemical production scheme, one would need to analyze even more holistically than in this review or the contribution by Vogt and Weckhuysen, linking hydrogen production, CO_2_ capture and sequestration, renewable fuels, and circular chemical production. Mayer et al.^[^ ^7^ ^]^ recently published an excellent analysis of the conversion of the chemical industry with a focus on the integration in an overall energy system and timing of the transition, but covering less the technology components available and still needed for the transition.

**Figure 1:**
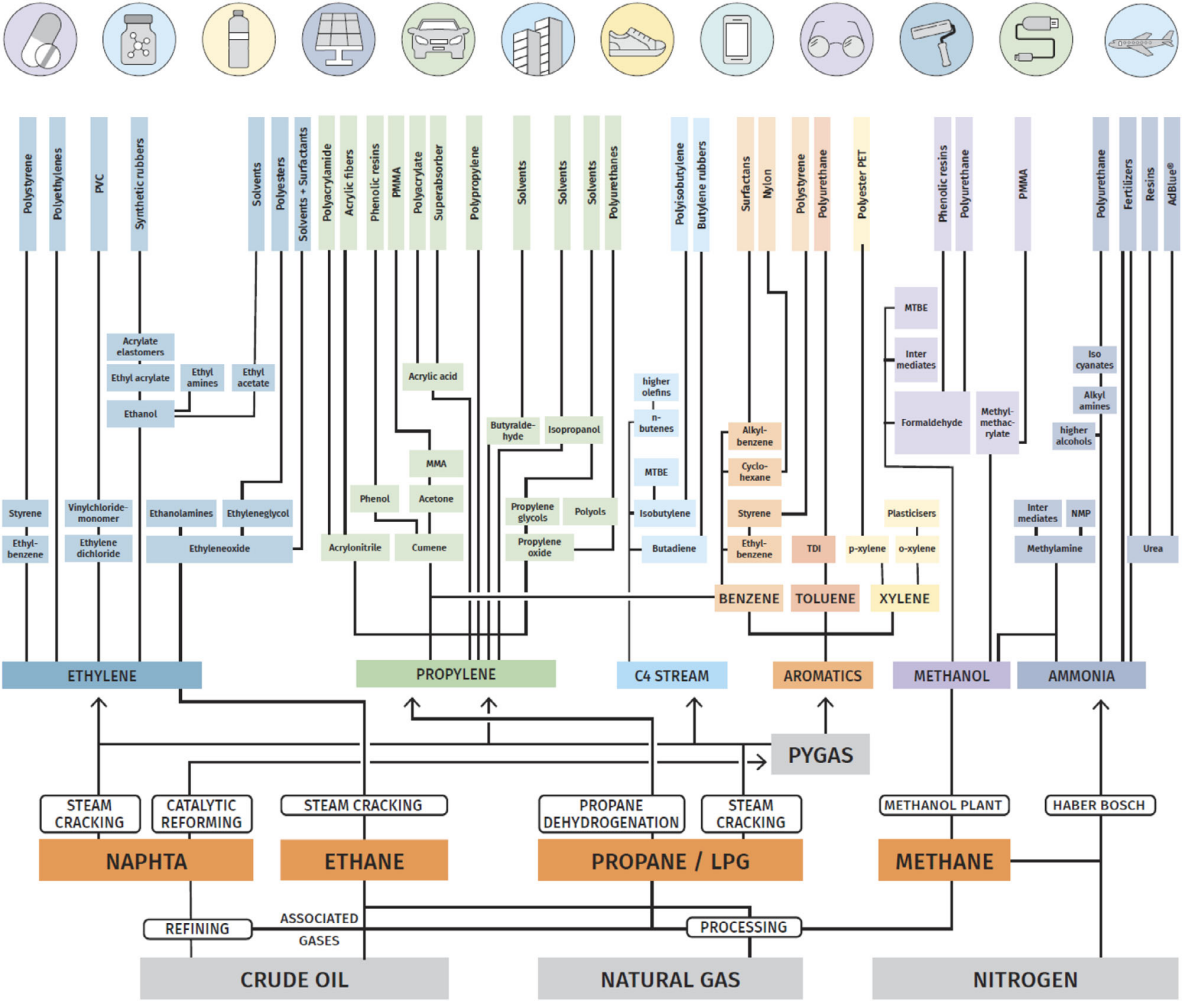
Simplified flowchart of carbon and nitrogen‐based products of the chemical industry (inspired by and simplified/modified from: https://www.petrochemistry.eu/about‐petrochemistry/flowchart/).

If one focuses on the chemical industry—which currently to a large extent piggybacks on the petroleum industry for carbon‐containing feedstocks as raw materials supply—there is a relatively small platform of molecules that provide the major material input into the chemical value chains (Figure 1). From these, intermediates are generated that are in turn converted—over a few or many steps—eventually to the tens of thousands of individual chemical products.

The molecules we use nowadays are almost invariably obtained from fossil resources, namely oil and natural gas, and in parts of the world also from coal. Thus, if chemical production should approach greenhouse gas neutrality, the whole basis has to be completely changed. Nowadays, the petrochemical industry relies on four very large volume input streams: (1) olefins (predominantly ethene, propene and butenes), (2) methanol, (3) aromatics (benzene, toluene, and xylenes), and (4) hydrogen (not explicitly shown in Figure 1, but used, for instance, for ammonia production and produced by methane reforming). Of course, there are other crucial compounds, which are needed, such as nitrogen for ammonia production and its downstream products, or heterocycles. However, these are either not associated with a substantial carbon footprint, if the process energy for production is renewable, are comparably much less important on a weight basis, or can be traced back to the other compounds already mentioned. If the input streams (1) to (4) could be kept, but produced without fossil resources, this would be a very solid basis for a substantial reduction of the carbon footprint of the chemical industry. One should keep in mind that an appreciable part of the carbon footprint of the chemical industry also results from the specific energy demand for production. Electrification of chemical production, based on renewable electricity, i.e., of heaters, compressors, and other equipment, is thus also a very important task.

In the following, the feedstock base will be discussed in more detail, treating hydrogen first, since all of the following products and processes crucially depend on the availability of cost competitive hydrogen obtained from sources with essentially zero greenhouse gas footprint. More detailed analyses are certainly necessary and are available for some compounds, such as for methanol synthesis via different pathways,^[^ ^8^ ^]^ but we predominantly want to line out the bigger picture. The majority of chemical production with respect to produced total mass results in different types of polymeric products (plastics, 413.8 Mt/a^[^ ^9^ ^]^), for which recycling ratios are still appallingly small. Increasing circularity of polymers would thus change the overall picture substantially. These aspects will be discussed in the latter part of this contribution. We will also not cover in detail the question how to source the CO_2_ as feedstock in a post‐fossil world. There still will be point sources providing large amounts, such as biogas‐plants or cement (clinker) and lime production. However, there are strong efforts for direct air capture of CO_2_ (DAC), for which first pilot plants are running. DAC technology is covered in various recent reviews, and interested readers are referred to those.^[^ ^10^, ^11^, ^12^, ^13^ ^]^

## 2 Hydrogen Without Greenhouse Gas Emissions

The vast majority of hydrogen today is produced from fossil resources, particularly natural gas, which is converted by steam reforming, autothermal reforming, partial oxidation, or dry reforming to first syngas, which is then further processed by the water‐gas shift reaction to hydrogen and carbon dioxide. The CO_2_ footprint of these processes is on the order of 10–12 kg CO_2_/kg H_2_.^[^ ^14^ ^]^ This also gives a first indication, how a future price of such fossil hydrogen is affected by CO_2_ pricing: the production cost of hydrogen over the last years was typically around 1–1.50 €/kg, strongly depending on natural gas prices, the major cost factor, and quite different in different parts of the world. The price of CO_2_ certificates in Europe has already been close to 100 €/t_CO2_, which would thus almost double the hydrogen price. A meta‐analysis of numerous papers on future hydrogen production costs by different technologies has recently been published by Frieden and Leker, and this study predicts that hydrogen prices may be around 2€/kg in 2050, with strong fluctuations for different regions of the world, but less strong differences between hydrogen obtained by electrolysis and methane steam reforming.^[^ ^15^ ^]^

Hydrogen with low CO_2_ emission footprint is available by different pathways^[^ ^16^ ^]^ which include electrolysis with renewable electricity (“green” hydrogen), reforming of fossils with capture and storage of CO_2_ (blue), or methane splitting with storage of solid carbon involving different kinds of energy for the splitting reaction (turquoise). Blue hydrogen is discussed as a viable short‐term solution, especially in the beginning of a transition, since for the next years, maybe decades, there will not be sufficient renewable electricity available at attractive cost for the production of sufficient amounts of green hydrogen. Blue hydrogen relies on technology components, which are essentially known at scale, even if massive global deployment would require further improvement. Carbon dioxide removal after the water‐gas shift step is also required in current hydrogen production schemes, and typically an amine wash is used, at costs of around 40 €/t.^[^ ^17^, ^18^ ^]^ The cost for the storage step has to be added. For geological storage, suitable geological formations are required. Such formations are abundant, and operational experience on the scale of almost a million tons per year is available from the Sleipner gas field, where CO_2_ has been injected into a sandstone formation for final storage over more than 20 years.^[^ ^19^ ^]^ A very informative review on carbon dioxide storage in different projects is given be Snæbjornsdottir et al.^[^ ^20^ ^]^

Green, together with turquoise, hydrogen, on the other hand, may be the long‐term more viable, possibly even the cheaper solution. Since most renewable power is harvested in the form of electricity, i.e., from photovoltaic systems or wind turbines, electrolysis seems to be the most straightforward production pathway. Four different schemes are available, i.e., alkaline electrolysis, acidic electrolysis (also called PEM, for proton exchange membrane, electrolysis), anion exchange membrane electrolysis, and high temperature electrolysis, with the latter two still being in the development phase. An excellent comparison between these technologies, including a comparative table, is available in a recent review.^[^ ^21^ ^]^ In addition, there are more advanced schemes, like plasma‐assisted approaches or co‐electrolysis systems with CO_2_, which could directly lead to syngas with the desired composition. However, these are at a much lower technology readiness level (TRL), and possibilities for practical deployment are uncertain. Thus, in spite of potential systemic benefits of such schemes, they will not be discussed here.

Alkaline electrolysis is most mature,^[^ ^22^ ^]^ with commercially available systems having capacities up to about 1000 m^3^ h^−1^. Electrodes consist typically of nickel or cobalt‐coated stainless steel; anode and cathode spaces are separated by a ceramic diaphragm. Alkaline electrolyzers have a limited dynamic range, which is a disadvantage when used with varying loads, as might be the case in renewable energy systems, and they only have limited pressure capabilities. This is different for PEM electrolyzers,^[^ ^23^, ^24^, ^25^ ^]^ which, at least for short periods of time, can operate between 5% and 300% of their nominal power rating and deliver hydrogen at elevated pressure at almost unchanged efficiency. The downside is their reliance on noble metal electrocatalysts, among them the scarce iridium and other platinum group metals, for the anode side. Scarce raw materials are certainly a problem, and recycling schemes to provide circular solutions are mandatory for these. On the other hand, for most technologies, there is a second/third/fourth… best solution. Alkaline electrolyzers operate reliably for decades, and advances in more flexible operations have been achieved.^[^ ^26^ ^]^ Moreover, there are strong efforts to replace the scarce and expensive metals with more abundant alternatives.

Eventually, cost of production will be the crucial point for large scale deployment of electrolyzer technology. There are two essential factors determining the cost, which are the electrolyzer Capex (systems level) and the cost of renewable electricity.^[^ ^27^ ^]^ A comprehensive analysis, comparing also cost for other hydrogen technologies by Matthes et al.^[^ ^28^ ^]^ comes to a similar conclusion concerning the key parameters of electrolysis hydrogen. While electricity prices of 1 €cent/kWh are currently unrealistic for wind and photovoltaics in northern and central Europe, bids for photovoltaic power plants with electricity prices substantially below 1 $cent have been submitted over the last years for the Middle East.^[^ ^29^ ^]^ If the capacity factors of electrolyzers would be increased by using additional sources of renewable electricity, hydrogen production costs as low as 2 €/kg (in one study even 1 €/kg is claimed^[^ ^30^ ^]^) could come within reach, which would bring the costs into the same price range as fossil hydrogen carrying a substantial CO_2_ penalty. The cost for at least part of the hydrogen produced by electrolysis could be additionally reduced by implementing a value‐generating anode reaction^[^ ^31^ ^]^ instead of production of oxygen, which is currently mostly released into the atmosphere without generating additional revenues. As a final point in this section, the water demand is sometimes brought up as an argument against the production of green hydrogen. While this certainly needs to be considered, especially in arid world regions, overall the demand for water as raw material for electrolysis is small compared to other uses.^[^ ^32^ ^]^ In addition, it could be sourced from seawater by using desalination plants, for which the energy demand is estimated at 3–6 kWh per cubic meter of water.^[^ ^33^ ^]^ A cubic meter of water would generate more than 100 kg of hydrogen, with an energy demand for electrolysis of around 5000 kWh, against which the energy required for the production of the feed water is negligible.

The discussion in the preceding paragraph only relates to the production cost of hydrogen. Green hydrogen would probably advantageously be produced in (remote) world regions with favorable solar or wind power, and needs to be transported to consumption centers, adding to cost and CO_2_ footprint.^[^ ^13^ ^]^ Several transport options are being discussed, such as in liquefied form (a first prototype shipment was delivered earlier in 2022 from Australia to Japan), or after transformation to a carrier compound, such as ammonia, methanol, or a liquid organic hydrogen carrier (LOHC). Of these, both ammonia and methanol have their own very substantial markets as chemicals, independent of hydrogen transport, ammonia with approximately 170 million tons per year in 2019^[^ ^34^ ^]^ and methanol with approximately 91 million tons per year in 2023.^[^ ^35^ ^]^ A future hydrogen transportation infrastructure could thus take advantage of the infrastructure required for the global use of ammonia for fertilizer production or of methanol as chemical feedstock. For both compounds, production facilities would probably be best placed where cheap hydrogen is available. There are studies, which come to the conclusion that it is in most cases favorable to produce the hydrogen regionally.^[^ ^36^ ^]^ Cheap electricity in remote parts of the world, such as in the Sahara desert by PV or in Patagonia by wind, allows cheap hydrogen production, but converting it to a transport vector to ship it to industrial consumption centers may offset the production cost advantage. On the other hand co‐development of a global transport infrastructure for a chemical commodity, such as ammonia or methanol, which is shipped around the world in any case, and its simultaneous use as hydrogen vector may change the picture. Overall, selecting the right transportation technology is not a simple question, since many parameters, such as storage density, energy requirements for conversion to the storage form and back to hydrogen, technological complexity, cost, technology readiness level and many others, need to be optimized. Discussing these questions would by far exceed the scope of this contribution, but there are studies available, in which this and related issues are discussed at depth, for instance by Wang et al.^[^ ^37^ ^]^

## 3 Methanol Without Greenhouse Gas Emissions

Methanol is currently the crucial building block for C_1_ compounds in the chemical industry, and is also discussed as a general basis for the chemical and energy industry.^[^ ^38^, ^39^, ^40^ ^]^ It is produced from syngas over Cu/ZnO/Al_2_O_3_‐catalysts. The syngas, in turn, is obtained by different types of reforming from fossil resources, mostly natural gas. Current methanol synthesis technology has a CO_2_ footprint of between 0.5 kg CO_2_/kg methanol, if natural gas is the raw material, up to about 3 kg CO_2_/kg methanol for production from coal;^[^ ^5^ ^]^ a very useful overview of current technology and advanced solutions, including techno‐economic analyses, is given by Dieterich et al.^[^ ^41^ ^]^ However, as long as the methanol is not incorporated in long‐lived products, the carbon in the methanol will eventually end up in the atmosphere as CO_2_, which would lead to an additional footprint of about 1.4 kg CO_2_/kg methanol.

**Figure 2:**
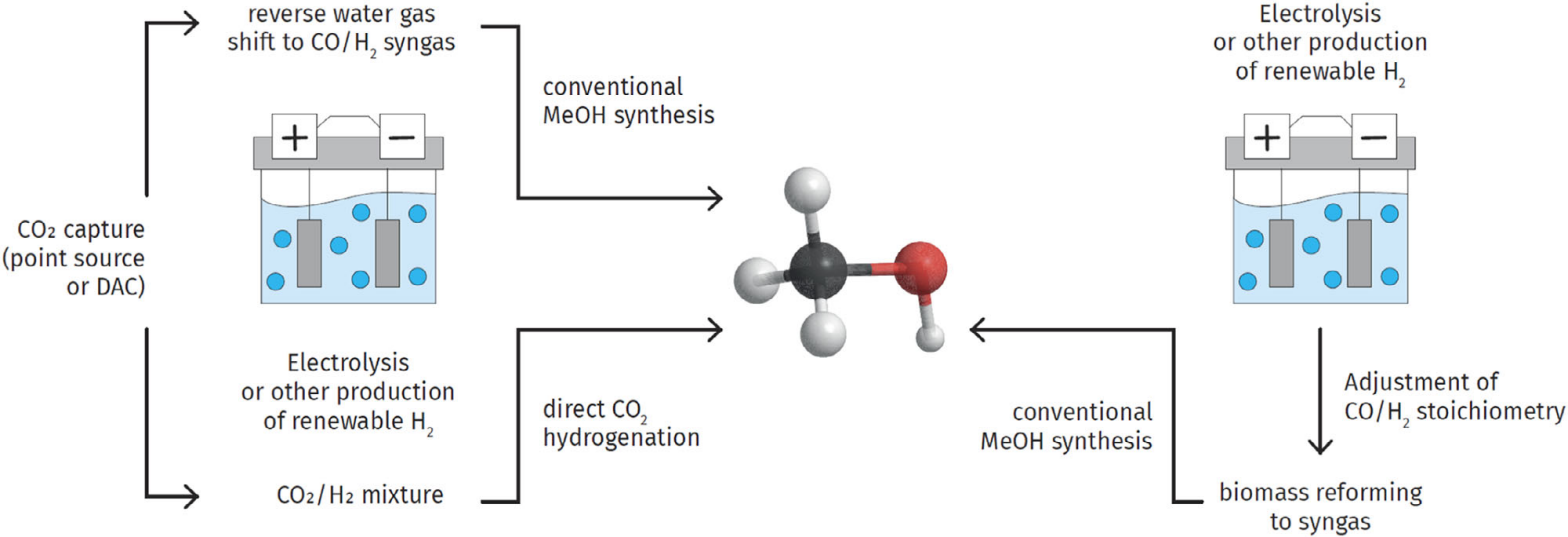
Pathways to sustainable methanol. CO/H_2_ stoichiometry of the product gas from biomass reforming can be adjusted with renewable hydrogen or by a reverse water–gas‐shift step.

Thus, in order to produce methanol with zero CO_2_ footprint, one would need to use a carbon source, which is CO_2_ neutral, and additionally, as much as possible, eliminate fossil energy input into the production process, i.e., drive heaters, blowers, etc. by renewable electricity. A schematic overview of production pathways is given in Figure 2. An interesting life cycle analysis (LCA), where methanol is synthesized from CO_2_ captured from a coal‐fired power plant and electrolysis hydrogen, claims negative emissions, i.e., −0.752 kg CO_2eq_/kg methanol.^[^ ^5^ ^]^ It has to be noted, though, that the negative emissions only result from the credit for avoiding some of the emissions of the power plant. If one combines the power plant and the methanol plant, there are still substantial net CO_2_ emissions. The framework for this LCA includes current technology—and its associated greenhouse gas emissions—for CO_2_ capture, transport of various chemicals, and so on, which will be less greenhouse‐gas intensive in a fully defossilized world. Additionally, biomass‐based routes are reported to have low or negative emission footprints, but since competition for biomass might be strong in the future, relying directly on CO_2_ as a raw material appears as the safer option—at least as a fallback.

Starting methanol synthesis from CO_2_ alone poses substantial challenges. It relies either on direct CO_2_ hydrogenation or conversion of CO_2_ to CO via the reverse‐water‐gas‐shift (rWGS) reaction. Currently, both technologies are not yet available on the scale required to replace the approximately 100 million tons of methanol per year, let alone a higher demand created by larger scale use of the methanol‐to‐olefins (MTO) and other processes starting from methanol.

For the rWGS reaction, high temperatures are required for substantial single‐pass conversion due to thermodynamic limitations. Moreover, at temperatures below 500 °C, methane formation is thermodynamically the preferred reaction for almost any practically relevant inlet composition.^[^ ^42^ ^]^ Therefore, rWGS on technical scale will either need highly active and selective catalysts which essentially suppress methanation, or requires temperatures of above 800 °C. Especially the latter is associated with a number of challenges: (1) thermal management of the reactor has to be carefully optimized, (2) in order to avoid additional CO_2_ emissions, the heat needs to be supplied via greenhouse‐gas‐neutral means, and (3) any catalyst material may deactivate by sintering or coking under these conditions.^[^ ^33^ ^]^

There are numerous catalysts for rWGS, which have been discussed in the scientific literature,^[^ ^33^ ^]^ but there is still substantial potential for further development. Two main mechanisms are discussed: an associative mechanism and a redox mechanism, or variants thereof.^[^ ^43^ ^]^ In the redox mechanism, CO_2_ oxidizes oxygen defect sites on the catalyst, mostly on an oxide support, resulting in CO formation. The oxygen defects are recreated by reaction with hydrogen, typically spilled over from supported metal species. In the associative mechanism, an adsorbed carbonate‐species reacts with adsorbed hydrogen, an oxygen atom is abstracted, resulting in an adsorbed formate species, which decomposes to desorb CO. These mechanisms were developed for low temperature rWGS below 500 °C, where conversion is strongly equilibrium limited, but studies on methane dry reforming^[^ ^44^ ^]^ suggest that such a mechanism also prevails at higher temperatures. Knowledge concerning the mechanism is especially relevant in the low temperature range, since methane formation must be suppressed. For the high temperature rWGS, on the other hand, the stability issue of the catalyst is much more important, since thermodynamically conversion is high and CO is the main product. Thus, for further development, supported metals seem to be particularly promising for processes at lower temperatures, while highly temperature‐stable oxides, possibly also supported, generally seem to be more suitable for high temperature rWGS. However, other options, such as α‐Mo_2_C,^[^ ^45^ ^]^ could be interesting solutions. Given the importance of rWGS for CO_2_ valorization and the development status, one can expect that a process on a world scale can be developed over the next ten years. This will, however, require intensified efforts from both academia and industry, and cooperation between science and engineering – which has already started, since the number of publications on rWGS has increased essentially exponentially over the last years.

In specific situations, electrochemical conversion of CO_2_ to CO could be an attractive alternative. CO_2_ fed to the cathode of an electrolyzer can be reduced to CO, and together with the cathodically formed hydrogen, result in syngas as a product.^[^ ^46^ ^]^ A process for electrochemical CO_2_ reduction in a solid oxide electrolysis cell (SOEC) is offered commercially by Topsøe.^[^ ^47^ ^]^

The alternative for methanol synthesis from CO_2_ is direct hydrogenation to methanol without the detour via syngas. Generally, the Cu/ZnO/Al_2_O_3_ catalyst system is also suitable for methanol synthesis starting from CO_2_‐rich feeds—even in conventional methanol synthesis, the feed contains some CO_2_, the exact fraction depending on the process. However, the substantially higher water concentration resulting from CO_2_ as feedstock reduces catalyst activity and poses a more severe strain on the catalyst lifetime. Nevertheless, pilot plants and also the first production plant built by CRI in Iceland in 2012 on a scale of 1300 t/year (expanded to 4000 t/y in 2014) are based on the classical methanol synthesis catalyst, possibly somewhat adapted to the CO_2_‐rich feed, with no details disclosed.^[^ ^32^ ^]^ Currently, the 110 000 t/year Shunli plant is the biggest plant in operation, also built by CRI, and fed with captured CO_2_ and hydrogen from a coker. The plant was commissioned in the second half of 2022 and inaugurated early in 2023.^[^ ^48^ ^]^ While this plant is about a factor of 10 smaller than the world‐scale methanol plants based on conventional syngas feeds, the size is in a dimension that places sustainable methanol production from CO_2_ within reach.

The classical methanol synthesis catalyst system has been developed further for CO_2_‐rich feeds by the use of promoters, such as noble metals^[^ ^49^ ^]^ or zirconia and other oxidic promoters.^[^ ^38^, ^50^ ^]^ Also, the ZnO, a crucial component of the conventional methanol synthesis catalyst, has been replaced by other oxides, most notably by ZrO_2_, which is active both for CO_2_ and CO hydrogenation.^[^ ^51^ ^]^ Later developments show that also indium and noble metal‐free systems based on Cu/Ga/AlO_x_ are highly competitive and offer a cheap alternative.^[^ ^52^ ^]^

In more recent studies the copper of the conventional catalyst is completely replaced. A very useful overview is given by Guil‐López et al.^[^ ^53^ ^]^ The most interesting developments, which have the potential to eventually surpass the Cu/ZnO‐based systems for CO_2_ hydrogenation, contain indium, either as intermetallic compound with palladium or as indium oxide, together with other oxides. PdIn intermetallic compounds outperformed the conventional Cu/ZnO/Al_2_O_3_ catalyst over a wide range of conditions;^[^ ^54^ ^]^ however, the reaction was carried out in liquid phase, for which the conventional catalyst is not optimized. A highly promising system is the In_2_O_3_/ZrO_2_ catalyst.^[^ ^55^ ^]^ The indium‐based catalysts require higher temperatures than the conventional system, but selectivity to methanol remains essentially 100% over the whole temperature range studied up to 300 °C. Activity was retained over 100 h on stream, while the copper‐based system deactivated strongly—not surprising at the high temperature. Another, somewhat unexpected, alternative is MoS_2_. One would on first sight expect short lifetimes due to catalyst deactivation by water, but the catalyst was found to be stable over 3000 h at activities substantially exceeding both the Cu/ZnO based system and indium‐based catalysts.^[^ ^56^ ^]^

Given the progress achieved over the last years, both in the direct one‐step hydrogenation of CO_2_ to methanol and in the indirect process via rWGS, followed by conventional methanol synthesis from syngas, large scale implementation of sustainable methanol synthesis appears to be in reach. Dieterich et al.^[^ ^32^ ^]^ also provide a comparison of different socio‐economic analyses of sustainable methanol production, and while the costs for methanol depend strongly on the assumptions entering the analyses, the estimated price ranges are at least in the same order of magnitude as for conventional methanol, albeit in most studies somewhat higher. In a most recent analysis, the cost of fully renewable methanol was calculated to be about a factor of two higher than for conventional methanol for a 4000 t/a plant, with the electricity cost (assumed at 57.8 €/MWh) and electrolyzer capex having the highest potential to reduce this cost.^[^ ^57^ ^]^ With the CO_2_ emission certificate price increasing and both electricity costs for renewable electricity in favored parts of the world and electrolyzer capex decreasing, it is expected that the gap between fossil and renewable methanol will become substantially smaller over the next decades.

## 4 Producing the Olefins Basis

If methanol is available via an essentially zero CO_2_ pathway, also a straightforward route to sustainable olefins is open, i.e., the methanol‐to‐olefins (MTO) route,^[^ ^58^ ^]^ which can be complemented by other methods for olefins productions, such as ethanol dehydration^[^ ^59^, ^60^, ^61^ ^]^ or others discussed further below. The MTO process relies on the conversion of methanol to mostly ethylene, propylene and different C_4_ olefins over microporous silico‐alumo‐phosphate catalysts, especially SAPO‐34, resembling a classical zeolite. Instead of tetrahedrally oxygen‐coordinated, corner‐sharing aluminum and silicon atoms, in a SAPO the basis is a tetrahedrally oxygen‐coordinated aluminum/phosphorus framework (which would be neutral), in which part of the tetrahedral atoms is replaced with silicon. This leads to a negative framework charge, if predominantly phosphorus atoms are substituted. The pore structure of SAPO‐34, with rather small pores, restricts the formation of bigger hydrocarbon molecules and thus shows a particularly high selectivity for ethylene and propylene; additionally, C_4_ hydrocarbons are formed to a somewhat smaller extent.^[^ ^62^ ^]^ There are three major process variants of the MTO process:^[^ ^51^ ^]^ The first process was developed by UOP in collaboration with Norsk Hydro,^[^ ^63^ ^]^ later improved with respect to small olefins yield by coupling with an olefin cracking process of UOP and Total Petrochemicals. This process relies on a fluidized bed technology to facilitate thermal management and allow continuous regeneration of the H‐SAPO‐34 catalysts, which suffers from rapid deactivation by coke deposition. The process is implemented, for instance, in the Jiangsu Sailboat plant in China with a production capacity for ethylene/propylene of 833 000 tons per year. Already in 1997, a publication claimed better economics for the MTO process compared to steam cracking under certain conditions.^[^ ^52^ ^]^ Another variant of the MTO process, the DMTO process, relies on technology of Dalian Institute of Chemical Physics.^[^ ^47^ ^]^ The process proceeds from dimethylether or methanol, but relies on an H‐SAPO‐34 catalyst as well. Selectivity to ethylene/propylene can be improved by recycling of C_4+_ compounds to above 85%. Also the DMTO process uses a fluidized bed reactor and continuous catalyst regeneration. This process is commercially implemented in a number of plants with capacities of several hundred thousand tons of olefins per year.^[^ ^47^ ^]^ A final process variant, the Lurgi methanol‐to‐propylene (MTP) process, maximizes the propylene yield by recycling all other hydrocarbon products, and eventually propylene—and gasoline as byproduct—are obtained.^[^ ^64^, ^65^ ^]^ This process differs substantially from the other two, since here a ZSM‐5 catalyst is used, and it relies on parallel fixed‐bed reactors, in which periodic regeneration is possible, and temperature control and regeneration are achieved by the recycle and feed injection between beds. For MTP there are several commercial installations at capacities of several hundred thousand tons of propylene per year. Thus, a wide range of ethylene to propylene ratios and also the current C_4_‐basis could be provided by judicious choice of parameters, judging from selectivities for different products reported in different publications.^[^ ^47^, ^51^ ^]^

Moreover, the MTO process variants could be further supplemented by bioethanol dehydration, which would provide an alternative access to ethylene via a low CO_2_‐footprint route.^[^ ^48^, ^49^, ^50^ ^]^ Availability of bioethanol could increase in the future with the reduction in the number of gasoline‐powered cars, which would make redundant part of the ethanol admixed to the gasoline. The reaction is fairly straightforward, with the dehydration typically catalyzed by acidic solids, such as aluminas, zeolites, zirconia, magnesium oxide, and others. Under the right conditions, the reaction reaches selectivities of 95% and higher.^[^ ^48^ ^]^ The reaction proceeds between 300 and 500 °C at atmospheric pressure or slightly above. The reaction is endothermic, and thus good heat transfer into the bed is required in case of a fixed bed reaction; alternatively, fluidized bed reactors have also been described.^[^ ^48^ ^]^ For the biomass‐ethanol‐to‐ethylene process, a rough economic analysis resulted in competitive costs compared to steam cracking, depending on boundary conditions.^[^ ^48^ ^]^ For the combination with metathesis to provide propylene, costs for 2016—the year of the study—were calculated to be about 50% higher than for fossil propylene, but with substantial cost reduction potential.^[^ ^66^ ^]^ In Brazil, with its well‐developed bioethanol industry, commercial operation of such processes eventually leads to bio‐based polyethylene or ethylene glycol, which is then converted with terephthalic acid to polyethylene terephthalate.^[^ ^67^ ^]^ This last reference also gives a good account of the history of the ethanol‐to‐ethylene dehydration.

Further flexibility in the olefin mix can be achieved by ethylene dimerization, for which a commercial process is available,^[^ ^68^ ^]^ or by tuning the fractions of the different olefins by adding a metathesis step. It is based on the equilibrium between ethylene and 2‐butene on the one hand and propylene on the other. Such processes—the first one being the Phillips Triolefin process^[^ ^69^ ^]^—have been developed for adaptation of the olefin supply from fossil resources to market demand already more than 50 years ago. Catalysts are the typical metathesis systems, i.e., early transition metal compounds supported on oxides such as WO_x_/SiO_2_, often combined with isomerization catalysts, such as MgO, or other additives, such as phosphoric acid.^[^ ^70^ ^]^

**Figure 3:**
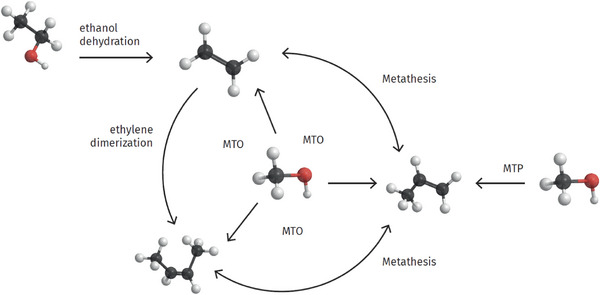
Current commercial pathways for the production of renewable ethylene, propylene, and butenes (MTO = methanol‐to‐olefins, MTP = methanol to propylene).

If such processes are combined, the fractions of different olefins via CO_2_‐neutral routes are adjustable at will (Figure 3 for an overview). Economics depend on many different factors, such as local feedstock situation and prices for CO_2_ emissions for fossil‐based production. A recent techno‐economical analysis for the UK (similar to other northern European countries) relying on wind power and CO_2_ sourced by direct air capture came to a cost of “green” ethylene of about 3000 €/t to 6000 €/t, depending on the exact process route—albeit at rather different technology readiness levels—with methanol synthesis/MTO being the most favorable one.^[^ ^71^ ^]^ However, this is approximately a factor of three more expensive than current ethylene market prices. In order to bring the cost into the range of ethylene from a steam cracker, especially the cost of electricity would need to be drastically reduced. Only then does it appear that the cost of olefins without substantial CO_2_ emissions can reach the same range as the cost of fossil‐based olefins.

There are emerging technologies, via which CO_2_ could be directly converted to olefins, both via thermocatalytic routes^[^ ^72^ ^]^ as well as by electrochemical CO_2_ reduction.^[^ ^73^ ^]^ However, the TRL of these routes is relatively low, and it seems currently improbable that they could develop to be competitive with the already commercialized processes mentioned above.

## 5 The Aromatics Platform

The aromatics platform consists primarily of benzene, toluene, and xylenes (benzene 2022 around 60 Mt,^[^ ^74^ ^]^ toluene 2022 around 30 Mt,^[^ ^75^ ^]^ xylenes around 50 Mt^[^ ^76^ ^]^); in addition, ethylbenzene is an important aromatic feedstock of the chemical industry, the majority of which is produced by ethylation of benzene. With respect to their end use, p‐xylene and ethylbenzene are the most important aromatic compounds, since they are used for the production of the mass polymers polyethylene terephthalate and polystyrene.

In the conventional chemical production routes, the aromatics platform is mostly obtained from petroleum, with raffinate (after reforming in the refinery) and pyrolysis gasoline (a side product of steam cracking of hydrocarbons) as the major sources. Sustainable access to aromatics is possible via different routes. With suitable catalysts and under the right operation conditions, methanol can be converted fairly selectively to aromatic molecules via the so‐called methanol‐to‐aromatics (MTA) processes, covered in an excellent recent review.^[^ ^77^ ^]^ MTA is still in a less advanced state than MTO, although a first pilot/production plant with a capacity of 30 000 t/a has been in operation since 2013; however, with the methanol feed produced from coal.^[^ ^78^ ^]^ The long‐known methanol‐to‐gasoline (MTG) process results in an aromatics fraction of up to 40% over the regularly employed acidic zeolite catalysts. In order to improve aromatics selectivity, bifunctional catalysts, often Zn‐ or Ga‐modified acidic zeolites, are used, with the additional functionality responsible for dehydrogenation and cyclization. Understanding and, based on this, improving catalysts can start with the knowledge gained from the much longer pursued MTO and MTG processes. Production of the olefins and hydrocarbons precedes aromatics formation, which requires dehydrogenation and cyclization. While zeolites—H‐ZSM‐5 is used in most studies—alone already produce a substantial fraction of aromatics from a methanol feed, economically competitive amounts require an additional dehydrogenation function in the catalyst, which is mostly introduced by Zn‐species, less frequently by Ga‐compounds. However, dehydrogenation comes at an expense, since it favors coke formation; aromatic species in themselves are considered to be coke precursors. MTA thus has to strike a good balance between aromatic yield and low deactivation. In order to improve the MTA process, Li et al.^[^ ^73^ ^]^ propose a combined strategy: fine‐tuning of metal sites and zeolite acidity as well as optimizing reaction conditions for improved aromatics selectivity, and tuning of zeolite crystal size and introduction of mesopores, as well as co‐feeding of specific hydrocarbons for extended catalyst lifetime.

In addition to changes in the catalyst, the process also needs to be substantially modified compared to the MTG process. In the Tsinghua University/China Huadian Corporation process implemented at the 30 000 t/a scale, a first reactor, operated at 450–500 °C, converts methanol to a mixture of aromatics and paraffins. Since the major target products are the xylenes, benzene, and toluene, together with the paraffins, are recycled back to the reactor, while the xylenes are separated. In a second reactor operating at about 100 °C higher temperature, the lighter hydrocarbons are converted to aromatics under the more severe conditions. The process provides quite some flexibility to favor the production of a desired product mix, with substantial future development potential, both with respect to the catalyst^[^ ^63^ ^]^ and reaction technology.^[^ ^64^ ^]^ Comparative economics of the process are difficult to predict due to its early development stage, but as for the other processes discussed, one can assume that decreasing prices for renewable methanol and increasing prices for fossil feedstocks due to higher CO_2_ pricing may favor such processes in the long run.

Also, the direct production of aromatics from syngas could be suitable by combining methanol synthesis and MTA in one reactor containing different catalysts for the different steps. This is discussed and analyzed by Li et al.,^[^ ^63^ ^]^ who conclude that aromatics formation is generally possible via an FT‐type olefin intermediate or via the methanol route. Both pathways entail their own challenges, and at this point, it is unclear whether such integrated processes or dedicated MTA plants would eventually provide the most efficient and cost‐competitive solution.

**Figure 4:**
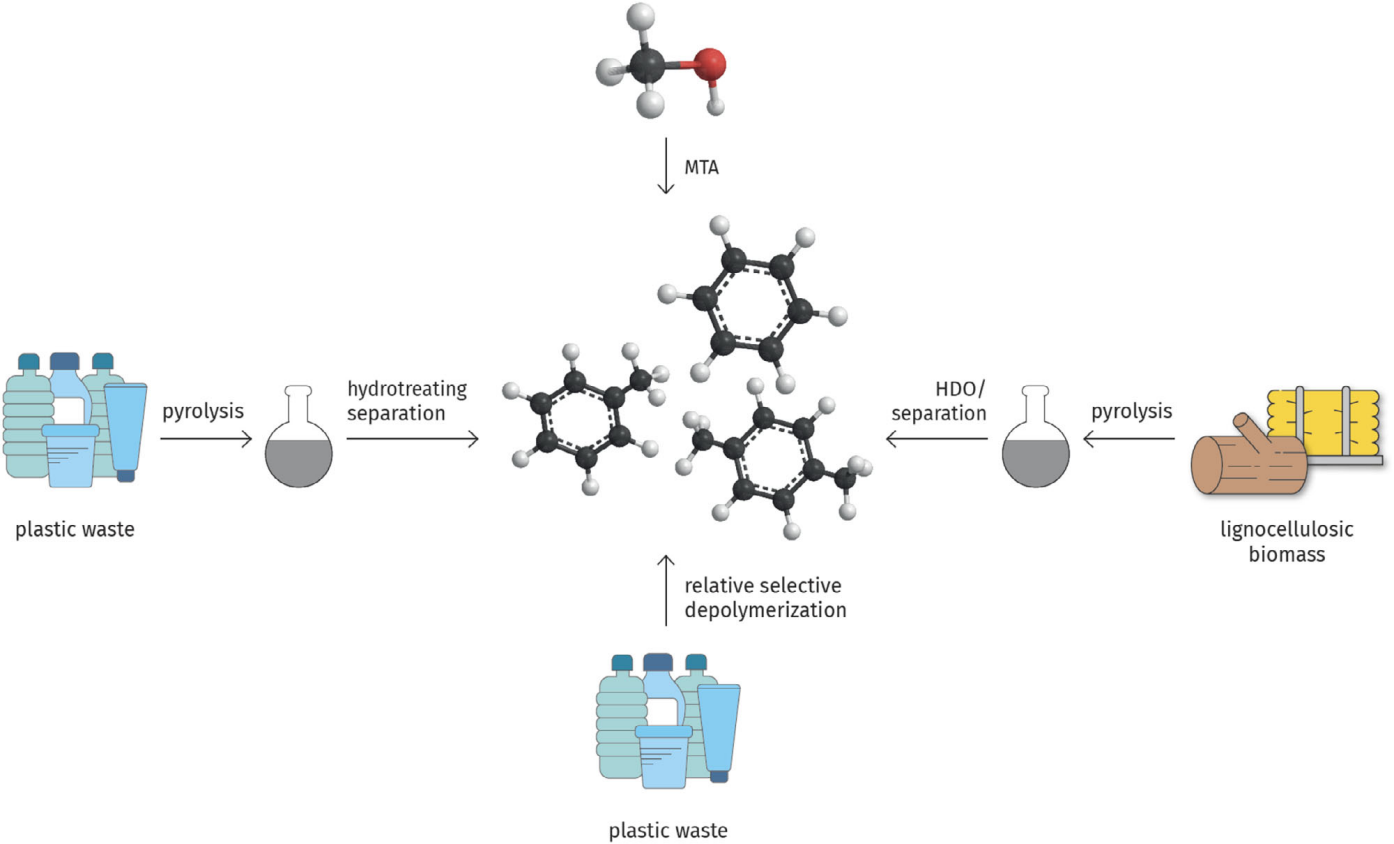
Major potential pathways for the production of sustainable BTX aromatics, starting from methanol, biomass, or plastic waste as raw materials.

However, as an alternative to aromatics production from C_1_ building blocks, nature provides high amounts of aromatic scaffolds in the lignin fraction of lignocellulose. Depending on the exact nature of the biomass, lignin comprises between about 15% and 40% of the lignocellulose. Global lignin production in the pulp and paper industry is estimated at about 70 Mt/a,^[^ ^79^ ^]^ but due to the oxygen content of lignin of about 30%, this corresponds to less than around 50 Mt/a theoretical yield of aromatic hydrocarbons, and practical yields of aromatic hydrocarbons from lignin are much lower still. Thus, lignin as the by‐product from the pulp and paper industry would be able to cover only much less than half of the current aromatics demand of the chemical industry, the rest would need to be produced either from recycled plastics or via one of the processes discussed above. Figure 4 summarizes the more important routes for the production of sustainable aromatics.

In addition, the production of clean aromatics from lignin is not straightforward. Lignin is a complex, highly cross‐linked biopolymer, with p‐cumarylalkohol, coniferylalkohol, and sinapylalkohol as major constituents, which are linked by aromatic ether bonds and C─C bonds in a rather irregular manner. Thus, in order to produce simple aromatic building blocks from lignin, hydrodeoxygenation and hydrogenolysis reactions are required, but under conditions so that aromatic rings are not hydrogenated. Moreover, the highest volume of lignin available on the market is Kraft‐lignin, which is characterized by a relatively high concentration of recalcitrant C─C bonds. It also contains rather high amounts of sulfur, which is a poison for many typical hydrogenation catalysts.

For lignin valorization, a first step is typically lignin depolymerization, which can proceed directly from the solid lignin, as shown by Romanenko et al.,^[^ ^80^ ^]^ or via production of a pyrolysis oil. The pyrolysis oil is a complex mixture of aromatic and aliphatic, highly oxyfunctionalized compounds. In both cases, hydrodeoxygenation reactions are key to stabilize the initial, depolymerized products and to produce the desired BTX aromatics.

However, currently the lignin fraction of lignocellulose biomass is typically considered the low‐value part of the feedstock, since the holocellulose part is relatively straightforwardly first converted to pulp, paper, and potentially second‐generation bioethanol. The effect of holocellulose extraction on the quality of the residual lignin is thus typically not considered to be highly important, impeding optimal use of the lignin. Alternative approaches, often termed as “lignin‐first”,^[^ ^81^ ^]^ might change the overall economics of biomass use and eventually facilitate aromatics production from lignocellulose.

## 6 Ammonia Production

The annual global ammonia production is quoted at about 185 Mt/a (with some variation, depending on source). Modern plants require 27–36 GJ/t_NH3_ of energy, corresponding to about 1% of the total man‐made greenhouse‐gas emissions. By far the biggest contribution to ammonia production is the synthesis of hydrogen from fossil resources, in most cases from natural gas.^[^ ^82^ ^]^ Therefore, replacing fossil‐based hydrogen with CO_2_‐neutral hydrogen is crucial. The major remaining items in the overall synthesis are nitrogen generation—in conventional Haber–Bosch synthesis from the air used for autothermal reforming—and energy for compression and heating.

Cryogenic air separation or pressure swing adsorption (PSA) are the most cost‐effective methods for nitrogen supply, with cryo‐separation having advantages at high production volumes, PSA more in the medium size range, also depending on the required oxygen impurity level.^[^ ^83^ ^]^ Process energy for both processes could be supplied by renewable electricity, and thus with very low CO_2_ footprint. Also, the rest of the energy supply for blowers etc. could essentially be based on renewable electricity. In a careful study it was estimated that electrified ammonia synthesis under today's conditions could reduce the CO_2_ footprint to 25%–35% of that of the natural gas‐fed Haber–Bosch process.^[^ ^68^ ^]^ This is probably an upper bound, since the CO_2_ footprint of construction materials, etc., will decrease to much lower values in a fully renewable energy system in the future. In addition, generally altered process designs could open new opportunities. Ammonia production with a strongly reduced greenhouse gas footprint appears to be feasible, at costs that are at least in the same range as for the methane‐fed ammonia synthesis.^[^ ^84^, ^85^, ^86^ ^]^

In addition, there is currently renewed interest in new catalysts and processes for ammonia synthesis. The groups of Hosono and Kitano, for instance, have developed a series of catalysts with low‐temperature ammonia synthesis activity.^[^ ^87^ ^]^ There are also recent reports on room temperature/atmospheric pressure mechanocatalytic routes, either directly or via chemical looping, albeit at low productivity.^[^ ^88^, ^89^, ^90^ ^]^

## 7 Waste Plastic as Feedstock

Treating plastics recycling in appreciable depth would by far exceed the scope of this contribution, and there are various interesting publications covering the effects of a circular—or more bio‐based—plastics economy on the carbon footprint of the industry.^[^ ^91^, ^92^, ^93^, ^94^ ^]^ While plastics are currently considered mostly as waste, they could become a highly interesting feedstock for the chemical industry in the near future. The amount of plastics annually produced was added up to around 400 Mt in a very useful recent review on plastics recycling^[^ ^95^ ^]^—which, when compared to the production amounts of base chemicals, incidentally shows that the majority of the platform molecules on a weight basis eventually end up in different types of polymers. Tapping into this resource, therefore, appears to be mandatory, with the added benefit that the pollution of land and oceans with plastic waste would be reduced.

Generally, there are two fundamentally different routes for chemical recycling of plastics: (i) relatively unselective (catalyzed) pyrolysis or (ii) selective depolymerization back to monomers or compounds similar to the original monomers. Which pathway is feasible and which process configuration exactly needs to be used depends on the type of polymer and the purity of the feedstock. This is, amongst other aspects, described in more detail in an excellent survey on the topic of chemical plastics recycling.^[^ ^96^ ^]^ Some polymers can relatively easily be decomposed back to the monomers. Polymethylmetacrylate (PMMA) or polystyrene, for instance, can be pyrolyzed to result in monomer yields exceeding 80%,^[^ ^74^, ^97^ ^]^ for PMMA reaching almost quantitative conversion at pyrolysis temperatures around 500 °C.^[^ ^98^ ^]^

Discussing different methods of depolymerization targeted at the reformation of the monomers would exceed the scope of this contribution, since the pathways are often rather specific. Readers are referred to the two reviews already mentioned,^[^ ^73^, ^74^ ^]^ which provide excellent access to the primary literature. Examples for such specific recycling routes are Nylon 6,^[^ ^99^ ^]^ for which BASF has announced a pilot plant,^[^ ^100^ ^]^ or polyurethanes.^[^ ^101^ ^]^ The more generally applicable method, which is also compatible with the feedstock supply of current chemical production, is pyrolysis to complex pyrolysis oils, which could be used as steam cracker feed to replace part of the current fossil feedstock. By employing suitable catalysts in pyrolysis processing, the product distribution can be shifted from predominantly aliphatic pyrolysis oils for the uncatalyzed case (can be converted to olefins in steam cracking) to predominantly aromatic products produced with different Y‐type zeolites, as demonstrated for the case of polyethylene pyrolysis.^[^ ^102^ ^]^ Zeolites of different structures and modified with different metals seem to be the most often used catalysts to direct the pyrolysis of polymer feeds to aromatic products.^[^ ^103^ ^]^

However, using waste plastic as feedstock for the chemical industry is complicated by many challenges. Currently, only a small fraction of plastic is globally collected and recycled in any form, estimated at 16% in 2018;^[^ ^73^ ^]^ most of the plastic ends up in landfills, incineration plants, or the environment. For targeted depolymerization, the waste would need to be separated to result in reasonably pure waste streams of specific polymers. If the waste is less pure and/or contaminated with non‐polymeric foreign matter, it would probably advantageously be converted to pyrolysis oil or gasified. The product stream (pyrolysis oil or syngas) would need to be purified to remove elements problematic in further processing, especially halogens, nitrogen, or metals, which are the most important heteroelements exceeding the specifications for current steam cracker feeds.^[^ ^104^ ^]^ Hydrotreating is generally a method for removal of heteroelements, but the hydrotreating methods developed in the petroleum industry—or for the treatment of biomass pyrolysis oil—cannot directly be used and have to be adapted to waste plastic feeds, for which further development is required.^[^ ^105^ ^]^ Moreover, novel technologies under development may provide alternative routes for plastics (and biomass) conversion, such as hydrothermal conversion using supercritical water.^[^ ^106^, ^107^ ^]^ In a very interesting and careful recent study, various different pathways for plastic recycling were compared in detail, with the conclusion, that by 2050 up to 60% of the naphta currently used in steam crackers could be replaced by recycled plastic, if the conversion chains are optimized. Going substantially beyond 70% naphta replacement, on the other hand, would come at an unacceptably high energy demand.^[^ ^108^ ^]^

## 8 Reducing the CO_2_ Footprint of the Energy Consumed in Chemical Production

The chemical industry also needs high amounts of energy for process heat and mechanical processes, for instance, for compression. It is estimated that more than half of the fuel consumption of the US chemical industry is used to provide process heat, mostly via steam, at different temperature levels.^[^ ^109^ ^]^ Compressors in the chemical industry are often also steam turbines, i.e., they ultimately rely on fossil fuel to generate the steam.

If the steam or direct process heat should be supplied with a negligible CO_2_ footprint, heat has to be generated by renewable electricity. Depending on the temperature levels available and required, heat pumps could serve as an efficient means, supplementing resistive heating to optimize the overall system. Compression energy could be supplied by electrically driven compressors.

As one example, BASF, together with Linde and Sabic, announced in 2024 the startup of a demonstration plant for an electrically heated steam cracker furnace relying on renewable electricity.^[^ ^110^ ^]^ This demonstration plant targets at a reduction of the CO_2_ emissions by more than 90%. Electrically driven cracking reactions, expanding the scope beyond steam cracking, are also comprehensively covered in a recent review article.^[^ ^111^ ^]^

Currently, large amounts of effluent heat from chemical plants are either not used or are fed into the steam grids as low‐pressure steam—a steam grade of limited value due to limited exergy content. Industry players have entered alliances to change this scenario and to valorize process heat better.^[^ ^112^ ^]^ It is expected that—especially for integrated chemical sites—massive savings can be achieved by the integration of adapted heat pump systems into chemical plants. For individual processes, heat pump integration has been analyzed, for instance, for coal‐to‐methanol,^[^ ^113^ ^]^ but also general frameworks to assess heat pump potential have been described.^[^ ^114^ ^]^ An even broader view of the integration of electricity in industrial processes has been given by Munoz‐Maldonado et al.^[^ ^115^ ^]^

Finally, the chemical industry is also well‐positioned to provide a balancing load for the electricity system. If the heat demand of the chemical industry is satisfied mostly by electricity, integrated heat storage systems would allow load‐leveling. Heat could be stored as steam at different temperature levels, but especially the chemical industry could use more advanced systems. For instance, hydrogen is available at most chemical sites. Many hydrides are known, for which the hydrogenation is exothermic, and the dehydrogenation is endothermic. Such systems could function as both hydrogen storage and heat storage units with high storage densities and at interesting temperature levels between 350 and 550 °C, depending on the choice of the metal. Magnesium operates at the lower end of the range, magnesium‐iron‐systems at the upper end,^[^ ^116^ ^]^ but with other hydrides, even wider operation windows are accessible.

## 9 Hydrogen Demand for the Conversion of the Chemical Industry to Greenhouse Gas Neutrality

Renewable hydrogen is a key component in a transformation process, which raises the question of the amount of hydrogen needed. The following rough estimate assumes that any chemical currently produced has to be synthesized from pristine raw material as discussed above, i.e., potential plastic recycling or biobased feedstock is not taken into account. Thus, the hydrogen stoichiometrically required to produce the key feedstock molecules (olefins lumped as ethylene, aromatics as benzene) will simply be added up as a first approximation:

Methanol is synthesized by the reaction

$${\text{CO}}_{2}+3H_{2}⇆{\text{CH}}_{3}\text{OH}+H_{2}O$$

Thus, for each ton of methanol produced, 0.1875 tons of hydrogen are required. Methanol currently enters the value chains of the chemical industry directly (100 Mt), and in a future system, it would additionally be required for olefin and aromatics production via MTO and MTA. In a simplified consideration just of ethylene, the MTO reaction proceeds as follows:

$$2\;{\text{CH}}_{3}\text{OH}⇆C_{2}H_{4}+2\;H_{2}O$$

for which each ton of olefins requires 2.28 tons of methanol, which at a demand of 255 Mt corresponds to an additional roughly 580 Mt of methanol for olefins production. Methanol to aromatics (benzene as proxy) proceeds formally as follows:

$$6\;{\text{CH}}_{3}\text{OH}⇆C_{6}H_{6}+6\;H_{2}O+3\;H_{2}$$

When calculating overall hydrogen demand, one has to take into account that the last reaction generates three molecules of hydrogen, corresponding to the formation of one methanol molecule, so that effectively only five methanol molecules are required for aromatics formation. Thus, for 100 Mt of aromatics via the MTA process, 205 Mt of methanol are necessary.

Thus, overall 885 Mt of methanol would annually be needed, corresponding to a hydrogen demand of 166 Mt per year. In addition, stoichiometrically, the 185 Mt of ammonia requires roughly 33 Mt of hydrogen annually, bringing the total hydrogen demand to about 200 Mt per year. This is a gigantic number. One ton of hydrogen by electrolysis requires about 50 MWh; this results in an additional electricity demand of 10 000 TWh. Current global electricity production is at about 25 000 TWh per year. Thus, converting the chemical industry to greenhouse gas neutrality using exclusively the pathways discussed above would require installing renewable electricity corresponding to 40% of the current global electricity consumption. For the example of photovoltaic electricity generation: if we use 2000 kWh m^−2^ as annual solar irradiation in sun‐rich parts of the world, and a solar cell efficiency of 20%, which is reached in commercial modules, then we would need 25 000 km^2^ solar panels to produce the corresponding annual amount of hydrogen. In order to achieve this by 2050, i.e., in about 25 years, one would need to install every single day from now to 2050 about 2.7 km^2^ new solar panels! This is not impossible; annual solar cell production capacity is expected to reach 1 TW (peak) per year in 2024,^[^ ^117^ ^]^ corresponding to a panel area of approximately 5000 km^2^, which exceeds this demand substantially, but it is still a tremendous challenge.

Of course, the above estimate only serves to illustrate the scale of the task; exact numbers will vary, depending on boundary conditions. But independent of exact numbers, the scale of the problem remains gigantic, and every year we wait to start the conversion process of the industry will make it much harder in the more distant future.

## 10 Outlook

**Figure 5:**
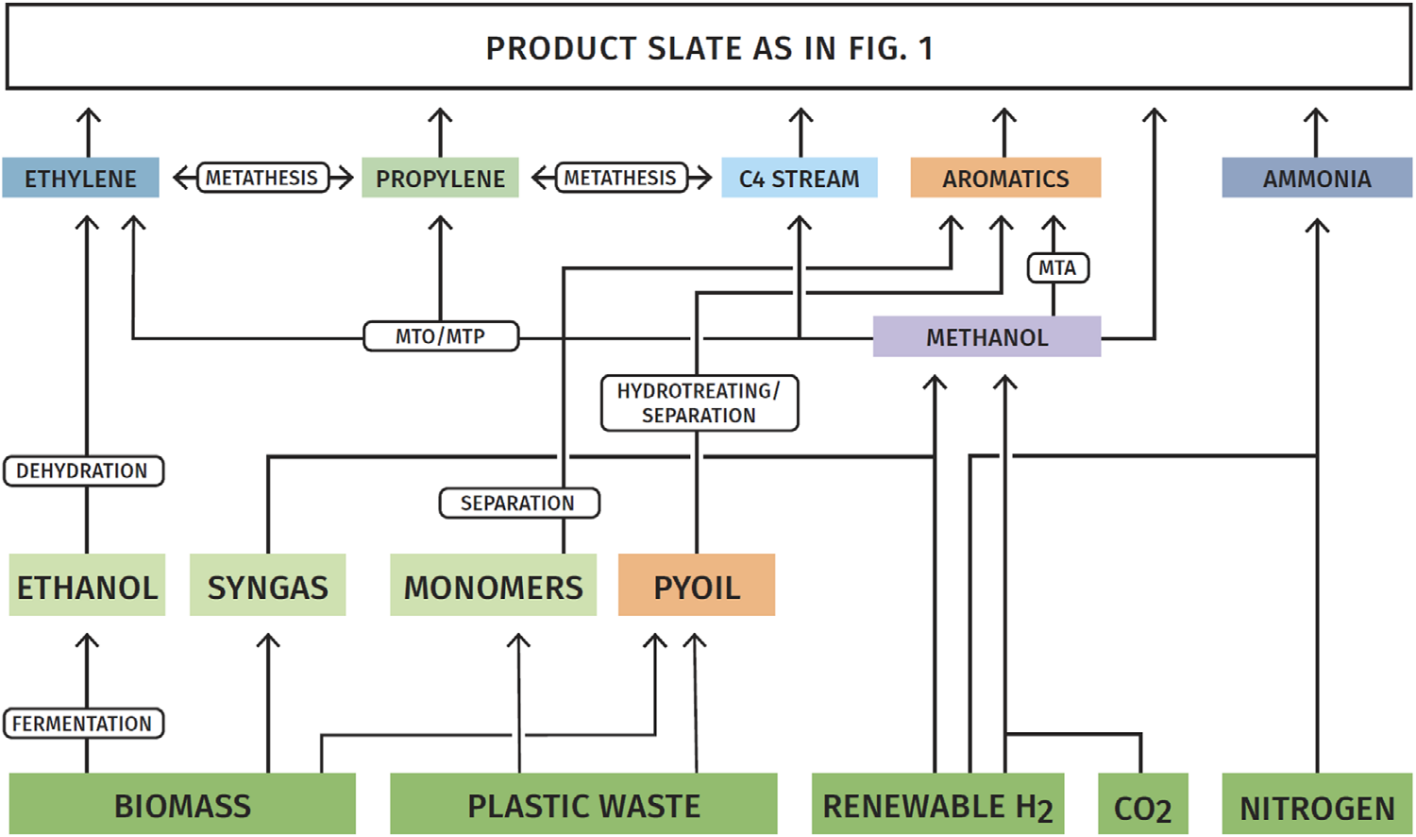
A renewable basis for chemical production. Not included is the whole slate of inorganic chemicals, which, however, have the biggest greenhouse gas footprint due to the energy input. If energy were sourced sustainably, these production processes could also be strongly defossilized (exception cement, see text).

The preceding discussion has shown that in general technologies are available or in development to replace the current fossil basis of the chemical industry by renewables and renewable energy, and at the same time maintain the majority of the technology basis of the downstream processes (Figure 5). Of course, for such a change more detailed life cycle assessments need to be carried out, and some of them have been referred to in this contribution. This is a major effort for the overall production chain, and there is rightfully increasing work invested in this task. Replacing the fossil basis will certainly come at a very substantial cost, since fossil resources are a cheap and convenient feedstock, at least as long as CO_2_ emission costs are low, but as technology proceeds and is being further developed, the cost gap will eventually close, at least for some production pathways. It is evident that the conversion of the chemical industry to a large part will rely on attractive pricing schemes for renewable energy and favorable regulatory framework for carbon dioxide removal, sequestration, and use—both topics which require support from political agendas. A wide range of instruments—of a regulatory nature or market‐based—has been suggested to reach effective and attractive boundary conditions, but these cannot be treated in detail here and the reader is referred to special publications for an overview.^[^ ^118^, ^119^, ^120^ ^]^ We will certainly also see alternative production processes further up the “chemical product tree”, such as dehydrogenation of ethanol to produce acetaldehyde using renewable ethanol,^[^ ^121^ ^]^ or novel electrochemical production pathways.^[^ ^122^ ^]^ In addition, the preceding discussion has predominantly focused on petrochemical products, which are essentially carbon based. The whole field of inorganic chemicals also needs to be addressed.

However, in this field, the major CO_2_ emissions do not result from the raw materials, but rather from energy for mining operations and process energy, which could be supplied from renewable sources. For instance, in metallurgical processes, carbon as a reducing agent could be replaced by hydrogen or directly by electricity, as difficult as this may be.^[^ ^123^ ^]^ There is one big notable exception, i.e., the inorganic materials obtained from carbonates, with the biggest product being cement. The CO_2_ released by the calcination of carbonate minerals can essentially not be avoided, and thus it has to be used as raw material, for instance, for methanol production (see above), or removed and prevented from entering the atmosphere by CCS schemes. Thus, there is more work to do than “just” the conversion, on which this contribution has focused, but removing fossil raw materials from chemical production is one very essential element.

## Conflict of Interests

The authors declare no conflict of interest.

## Data Availability

Data sharing is not applicable to this article as no new data were created or analyzed in this study.
